# Losing hope or keep searching for a golden solution: an in-depth exploration of experiences with extreme challenging behavior in nursing home residents with dementia

**DOI:** 10.1186/s12877-022-03438-0

**Published:** 2022-09-16

**Authors:** Annelies E. Veldwijk-Rouwenhorst, Sytse U. Zuidema, Martin Smalbrugge, Anke Persoon, Raymond T. C. M. Koopmans, Debby L. Gerritsen

**Affiliations:** 1 Department of Primary and Community Care, Radboud University Medical Center, Radboud Institute for Health Sciences, P.O. Box 9101, 6500 HB Nijmegen, The Netherlands; 2 Radboudumc Alzheimer Center, Nijmegen, The Netherlands; 3 Department of General Practice and Elderly Care Medicine, University of Groningen, University Medical Center Groningen, Groningen, The Netherlands; 4 Department of Medicine for older people, Amsterdam Public Health Research Institute, Amsterdam UMC - Vrije Universiteit, Amsterdam, The Netherlands; 5 De Waalboog “Joachim en Anna”, Center for Specialized Geriatric Care, Nijmegen, The Netherlands

**Keywords:** Challenging behavior, Dementia, Nursing home, Qualitative research

## Abstract

**Background:**

Situations of extreme challenging behavior such as very frequent and/or severe agitation or physical aggression in nursing home residents with dementia can be experienced as an impasse by nursing home staff and relatives. In this distinct part of our WAALBED (WAAL-Behavior-in-Dementia)-III study, we aimed to explore these situations by obtaining the experiences and perspectives of nursing home staff and relatives involved. This can provide a direction in providing tools for handling extreme challenging behavior of nursing home residents with dementia and may improve their quality of life.

**Methods:**

Qualitative multiple case study with individual interviews and focus group discussions. Interviewees were elderly care physicians, psychologists, care staff members, unit managers and relatives (*n* = 42). They were involved with nursing home residents with dementia and extreme challenging behavior living on dementia special care units in the Netherlands. For these residents, external consultation by the Centre for Consultation and Expertise was requested. Audio-recordings of the interviews were transcribed verbatim and analyzed with thematic analysis, including conventional content analysis.

**Results:**

Seven cases were included. Forty-one individual interviews and seven focus group discussions were held. For six stakeholder groups (resident, relative, care staff, treatment staff, nursing home staff, and the organization), three main factors could be identified that contributed to experiencing a situation of extreme challenging behavior as an impasse: 1) characteristics and attitudes of a stakeholder group, 2) interaction issues within a stakeholder group and 3) interaction issues among (groups of) stakeholders. The experienced difficulties with the resident’s characteristics, as well as suboptimal interdisciplinary collaboration and communication among the nursing home staff are remarkable. Nursing home staff kept searching for a golden solution or lost hope.

**Conclusions:**

This study offers important insights into situations of extreme challenging behavior in nursing home residents with dementia and offers caregivers targets for improving care, treatment and interdisciplinary collaboration, such as working uniformly and methodically.

**Supplementary Information:**

The online version contains supplementary material available at 10.1186/s12877-022-03438-0.

## Background

In nursing homes, over 80% of residents with dementia show challenging behavior, which encompass a broad spectrum of behaviors and become more severe as the dementia progresses [1–3]. A minority of cases consist of extreme challenging behavior, which is severe and/or occurs frequently [4–7]. In their seven-tiered model of the severity and prevalence of challenging behavior, Brodaty et al. categorise extreme challenging behavior as Tier 7, with an estimated prevalence described as rare [4]. In our WAALBED (WAAL-Behavior-in-Dementia)-III study, similar two-week prevalence rates of 7.4% of very frequent agitation, 2.2% of very frequent physical aggression and 11.5% of very frequent vocalizations were found [5, 6]. Previous studies have shown that challenging behavior has a great influence on the residents and their environment (relatives, nursing home staff and other residents), especially in case of aggression [8–10]. Extreme challenging behavior has an even greater impact on the resident, such as self-injury and the application of physical and chemical restraints, both influencing the quality of life of the resident negatively [11]. Furthermore, challenging behavior like severe physical aggression leads to injuries to other residents, which possibly influences their quality of life. Also, the extreme challenging behavior can lead to injuries, mental distress and even burnout among care staff or it can influence their decision to start looking for another job [8, 12]. The high impact of the behavior, together with its extreme severity and frequency, can lead to a situation in which an impasse is reached [4, 13], in which nursing home staff feels that they are out of (treatment) options and relatives feel powerless. This impasse is often preceded by a long trajectory of searching for the ‘right’ solution [14]. It is still unclear why a situation of extreme challenging behavior is experienced as an impasse by nursing home staff and relatives. To our knowledge, there is no theoretical framework in literature which already explains this. Although earlier literature describes that nursing home staff’s beliefs influence their attitudes, which in turn influence their response to the resident’s behavior itself [15, 16], the reasons why they experience a particular situation as an impasse are yet unknown but may be useful for breaking such a situation. Therefore, this qualitative study tries to answer the following research question: “Why are situations of extreme challenging behavior of nursing home residents with dementia experienced as an impasse by nursing home staff and relatives?” It aims to provide insight into experiences of nursing home staff (including their beliefs and attitudes) and to unravel contributing factors. Hereby we hope to provide tools for handling this behavior and to improve the care for and quality of life of nursing home residents with dementia and extreme challenging behavior.

## Methods

### Study aim, design, setting and participants

This explorative, qualitative study was performed as a distinct part of the WAALBED-III study that focused on nursing home residents with dementia and extreme challenging behavior [5, 6, 14]. Because of the lack of a theoretical frame to explain why situations of extreme challenging behavior in nursing home residents with dementia are experienced as an impasse, we decided to apply qualitative methods in this study. Hereby we were able to provide complex textual descriptions [17]. We used the Consolidated criteria for Reporting Qualitative studies (COREQ) to conduct and report the study. A detailed description of the applied methodology is presented in Supplementary material Table 1. In the Netherlands, care for people with dementia and extreme challenging behavior is mainly provided in dementia special care units by multidisciplinary teams of which the members are all employed by the nursing home (Table 1). In the following text, we will use the word nursing home staff for this, by which we mean the entire group of professionals. For this study, we included cases of nursing home residents with dementia and extreme challenging behavior for which external consultation from the Centre for Consultation and Expertise (CCE) [18] was requested. The CCE is a supplementary service to standard healthcare services which is funded by the Dutch government and provides expertise and support in the long-term care (including extreme challenging behavior) in people with dementia and intellectual disabilities. CCE works with independent experts in order to provide customized advice and support and accepts applications for consultation when there are serious concerns about a resident’s quality of life. Consecutive sampling was used to select cases, which means cases were selected in order of sign up according to their appropriateness for inclusion [19]. Cases were assessed for inclusion by two coordinators of the CCE, and by AV and DG through verification of the inclusion criteria: a) the resident had dementia and extreme challenging behavior which affected their quality of life according to the professionals who reported the case to the CCE; b) there was no obvious easily treatable cause for the challenging behavior; c) the behavior was experienced as very difficult to cope with by the involved nursing home staff and they had been unable to treat the challenging behavior satisfactorily; d) the challenging behavior consisted of aggression and/or vocally disruptive behavior and/or agitation; e) the resident had no acute life-threatening diseases; and f) they had been staying in the nursing home for at least 4 weeks. When a case was deemed appropriate for inclusion by the elderly care physician and the unit manager of the nursing home, intensively involved nursing staff members (as mentioned in Table 1) and the relative were asked for consent to participate in the study. For consent a written consent form was used.

**Table 1:** Setting of the study

**Members nursing home staff**	• Care staff: certified primary nurse assistants, nurse assistants, vocational trained registered nurses. • Treatment staff - elderly care physician - psychologist - physiotherapist - speech therapist - dietician - music therapist - occupational therapist • Unit manager: manager of the ward where the resident lives.

### Data collection

Several data were obtained from the residents’ medical files: demographical characteristics, duration of institutionalization, and prescribed medications. This explorative, qualitative study used individual interviews and focus group discussions to explore experiences of nursing home staff with situations of extreme challenging behavior of nursing home residents with dementia and to unravel contributing factors [17, 20, 21]. As mentioned before, these applied qualitative methods allow us to provide complex textual descriptions of how people experience a given research issue [17]. Topic lists for the interviews with professionals, relatives and focus group discussions were prepared by AV and discussed with the co-authors (see Supplementary Material Tables 2 and 3). The following topics were addressed: 1) nature and course of the behavior, 2) actions undertaken, 3) factors contributing to an impasse, 4) the impact of the situation on nursing home staff and relatives and 5) collaboration among nursing home staff. Six interviews per case were performed; one each with the involved elderly care physician, psychologist, certified primary nurse assistant, unit manager, another care staff member familiar with the resident, and one with a relative of the resident. The individual interviews with nursing home staff were held during April–December 2016 in the nursing home of the resident, while interviews with relatives took place during April–October 2016 at their own home (*N* = 4) or in the nursing home (*N* = 3). In addition, for each case a focus group discussion was held with the same interviewees of the individual interviews, except the relatives. Other care/treatment staff members could join the focus group discussion if they wished. The reason for performing six interviews per case and to conduct focus group discussions was to achieve data source triangulation and thereby to increase the validity and reliability of the results of the study [20, 21]. Moreover, with the focus group discussions we were able to collect a broad range of views, to examine the information obtained from the interviews and to further explore the cases [17]. The focus group discussions with nursing home staff were held during April 2016–January 2017 in the nursing home of the resident.

### Data analysis

All interviews and focus group discussions were transcribed verbatim and identifying information was removed. Transcriptions were analyzed with thematic analysis, an iterative process involving several steps [22]. This included conventional content analysis [23, 24] with the application of inductive coding (deriving codes from the data, modifying them throughout the coding process and providing an explanation of the data) and deductive coding (identification of potential categories and sub-categories as codes) [22] (AV, AP, EV, MW and KM). We coded on attributes and content [25]. For the attribute coding, we coded by (groups of) stakeholders: resident, relative, care staff, treatment staff (including unit manager), nursing home staff (care staff and treatment staff) and organization. We started with a thorough analysis of the first case to develop a viable procedure for subsequent coding. A coding tree was developed after grouping new codes into categories and combining them with existing codes and categories (AV, AP). After analysis of the first case, codes, categories and the coding tree were discussed in two separate meetings and a modified version was used for analysis of the other cases. During the coding process, the coding tree was altered. After analysis of the last case, the most recent version of the coding tree was used for re-coding the previous transcripts to improve accuracy of the analysis (MW). For within-case analyses, a mind map was created (EV, AV) and for cross-case analysis, a mind map constituting all other mind maps together was made (AV). Mind maps are “visual, non-linear representations of themes and sub-themes and their relationships” [26, 27]. For each case, consensus meetings took place with the data coders (AV, AP, EV, MW, KM) and one of the authors (DG). In all cases, AV was one of the coders. In these meetings, the mind map was discussed. All mind maps were further discussed in meetings with all of the authors. These discussions led to the refinement of categories into definitive main and sub-factors.

## Results

**Fig. 1:**
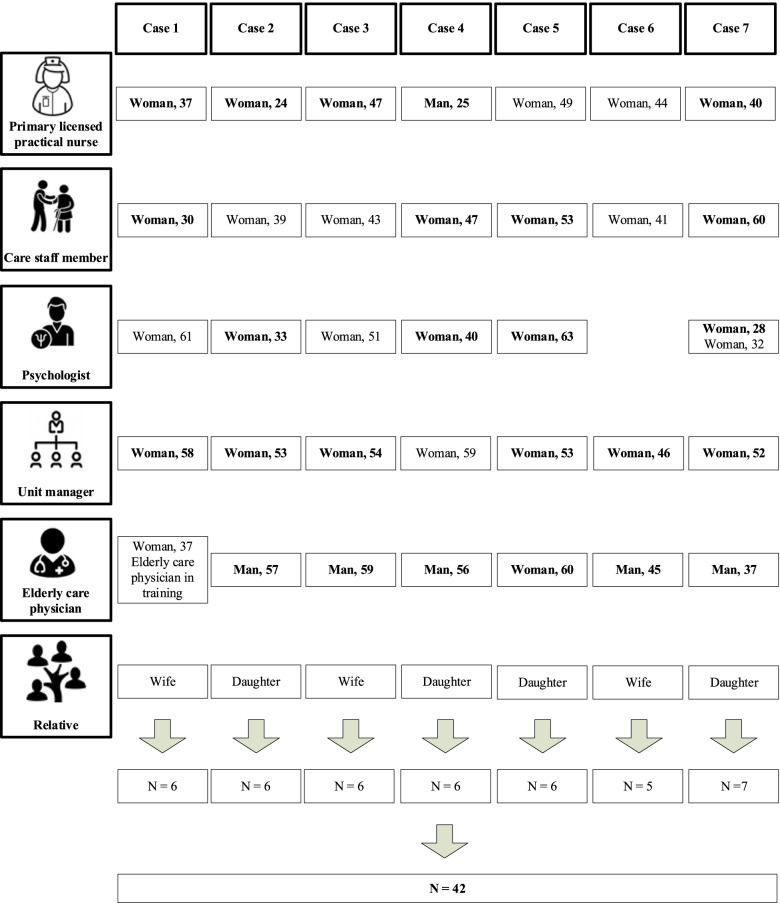
Characteristics of the included interviewees of the individual interviews (gender and age (years)). Notes: Interviewees depicted in bold type also participated in the focus group discussions

**Fig. 2:**
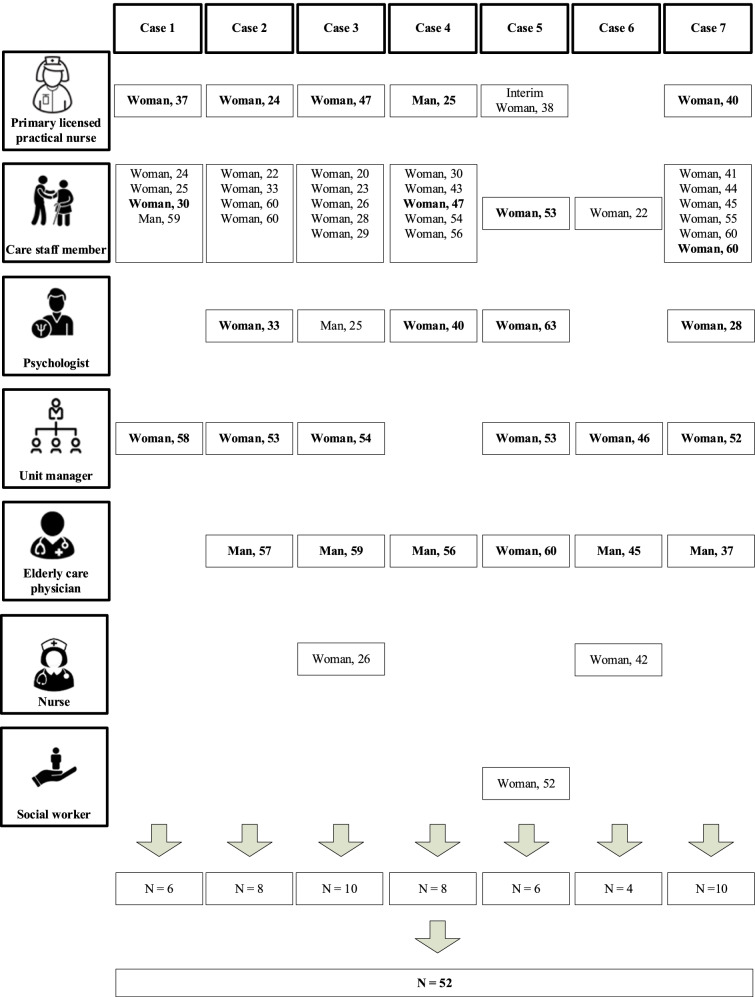
Characteristics of the included participants of the focus group discussions (gender and age (years)). Notes: Interviewees depicted in bold type also participated in the individual interviews

We expected to include ten cases, but stopped inclusion after interviewing for seven as we had reached data saturation, as determined by all authors. For the seventh case, no new codes were added to the coding tree [28]. We conducted 41 individual interviews with a total of 42 interviewees (one interview had two interviewees) and seven focus group discussions with a total of 52 interviewees (in six focus group discussions extra nursing home staff members attended who did not participate in the individual interviews). Background information of the interviewees is displayed in Figs. 1 and 2. The majority of the interviewees were women and their age varied between 20 and 63 years.

The challenging behavior of the residents consisted of extreme physical and/or verbal aggression and/or agitation (Table 2). Sometimes the behavior was unpredictable. Psychosocial interventions, as well as prescription of multiple psychotropics and, in certain cases, compulsory treatment had been applied to treat the challenging behavior.

*“Regarding care, every time you got hit, even though it was on your arm, you always got hit by him (the resident) …*. *There are several colleagues including me, who got truly hard blows in the face … Or a punch in the stomach.”* (Case 6, Certified primary nurse assistant in individual interview)

*“You are attempting all kinds of medication and ways of interaction with the resident … Well on a certain moment we have tried so many things all of us together. In addition, actually she (the resident) had all kinds of medication which you can prescribe for this kind of challenging behavior.. we tried so many things, also regarding psychological support, stimulating senses was tried very often in the living room … we have been so intensively involved with this behavior.”* (Case 7, Unit manager and elderly care physician in focus group discussion)

**Table 2:** Background information of each case

	Case 1	Case 2	Case 3	Case 4	Case 5	Case 6	Case 7
**Gender, age**	Man, 75 years old	Woman, 87 years old	Man, 78 years old	Woman, 89 years old	Woman, 89 years old	Man, 81 years old	Woman, 86 years old
**Duration of institutionalization**	22 months	21 months	18 months	24 months	27 months	29 months	18 months
**Department**	Psychogeriatric unit, small-scale	Psychogeriatric unit, small-scale	Psychogeriatric unit, small-scale	Psychogeriatric unit, small-scale	Psychogeriatric unit, small-scale	Psychogeriatric unit, large-scale	Psychogeriatric unit, small-scale
**Medical problems**	Parkinson’s dementia Morbus Parkinson Atrial Fibrillation Lower urinary tract symptoms	Dementia Heart failure Hypertension Osteoporosis Depressive disorder	Dementia with leukoencephalopathy Depressive disorder (2007) Rectal bleeding	Dementia Hip fracture (2016)	Vascular dementia COPD Heart failure Hypertension Atrial Fibrillation Cerebrovascular incident (2010) Recurrent urinary tract infections	Vascular dementia Hypertension Kidney failure Transient ischemic attack	Alzheimer’s disease Several transient ischemic attacks Hypothyroidism Osteoporosis
**Description of behavior**	Unpredictable moments of transgressive behavior and aggression (hitting, pushing, kicking, grabbing firmly) directed towards care staff and other residents	Yelling and screaming accompanied by fear and sadness Angriness	Verbal (yelling) and physical aggression (hitting, kicking, spitting, throwing with feces, squeezing breasts of care staff) directed towards care staff especially during personal care	Agitation Restlessness Attention seeking behavior (experienced as agitation) accompanied by fear and sadness Aggression directed towards care staff and other residents	Restlessness and yelling during the nights	Physical (hitting, kicking, grabbing) and verbal (cursing, insulting) aggression directed towards care staff, volunteers, family and other residents	Restlessness and angriness Beating on doors and windows Hitting directed towards other residents or care staff Slamming on tables Making noises
**Interventions described in the medical files**	**Family** Family consultation	**Family** Family consultation	**Family** Family consultation Involving family in behavioral consult	**Family** Making a voice recording with family	**Family**	**Family** Family consultation Visits from family member	**Family** Family consultation
	**Resident** Personal care with as little stimuli as possible and dosing stimuli by decreasing activities and offering more rest Relaxation massages Compulsory treatment (locking the door) Camera surveillance and emergency buttons on phones	**Resident** Offering rest (in living room) Changing the place at the table Offering personal attention, physical contact and safety by volunteers and trainees Daytime activities on a care farm Physical exercise (walking (outside)) Enclosure bed during the nights Accounting for the resident’s perception of the environment	**Resident**	**Resident** Offering adequate stimuli and familiar voices and noises Distraction Offering personal attention Giving foot baths	**Resident** Offering rest in the afternoon (in bed) Displaying picture of the resident’s wife in the resident’s room to create feeling of safety Giving tea or warm milk in the evenings Structurally notifying the resident of performed actions during care Structured day program Offering daily activities	**Resident** Personal care with as little stimuli as possible Offering rest (in the resident’s room**)** Structured day program Offering personal attention Offering daily activities as reading the newspaper, playing games Physical exercise (walking and swimming)	**Resident** Offering time-outs and rest by separating the resident from other residents Offering personal attention (also during meals) and physical contact Singing songs with the resident Handing a doll or a cuddle cat Offering multisensory stimulation (‘snoezelen’) Aromatherapy Transfer to another, quieter ward
	**Nursing home staff** Behavioral consult and crisis intervention plan by psychologist Self-defense course care staff Multidisciplinary team meetings Changing medication	**Nursing home staff** Behavioral consult and crisis intervention plan by psychologist Video-recordings of the behavior Advice regarding sitting comfortably Consulting internal consultation team External consultation psychiatrist Deployment of extra staff Moral deliberation session Changing medication	**Nursing home** **staff** Behavioral consult by psychologist Using wrist guards during personal care Training using video-feedback Observing colleagues during care provision Rotation of care staff Involvement of occupational therapist Multidisciplinary meetings Changing medication	**Nursing home staff** Behavioral consult by psychologist Expressive therapy Recruiting a nurse An employee working in the living room Deployment of extra staff Changing medication Drug holiday Applying intermittent palliative sedation	**Nursing home** **staff** Behavioral consult by psychologist Advice regarding stimuli Education and skill training of care staff about dementia and depression Changing medication	**Nursing home staff** Behavioral consult by psychologist and functional analysis of the behavior through the care programme ‘Grip on challenging behavior’ [29] Consult of an expressive therapist to be in line with the resident’s level of alertness Deployment of extra staff Changing medication	**Nursing home** **staff** Observation behavior and behavioral consult by psychologist Changing medication
**Relevant medication (name, dosage)**	Clozapine 25 mg bid Levodopa/carbidopa 50/12.5 mg bid Valproic acid 300 mg bid Clonazepam 0.5 mg as needed	Midazolam 15 mg qd Mirtazapine15 mg qd Oxazepam 10 mg qd Oxazepam 5 mg qd Oxazepam 5 mg as needed Venlafaxine 37.5 mg bid	Clozapine 50 mg qd Escitalopram 10 mg qd Mirtazapine 30 mg qd	Haloperidol 2 mg/ml 5drops qid Oxazepam 10 mg as needed Citalopram 10 mg qd	Levetiracetam 500 mg bid Oxazepam 10 mg as needed	Pipamperone 40 mg bid Citalopram 20 mg qd Memantine 5 mg qd Temazepam 10 mg as needed	Haloperidol 2 mg bid Citalopram 20 mg qd Pregabalin 75 mg bid Oxazepam 5 mg as needed

It appeared that factors contributing to experiencing these situations of extreme challenging behavior as an impasse could best be structured according to the six (groups of) stakeholders through attribute coding. Furthermore, three general factors could be identified using content coding: 1) characteristics of a stakeholder group, 2) interaction issues within a stakeholder group and 3) interaction issues with other (groups of) stakeholders (Fig. 1). For some (groups of) stakeholders, only one or two of these general factors were applicable. Moreover, numerous main factors and sub-factors could be identified. These factors are of a different nature; the general factors and main factors provide structure and are broad and overarching, the sub-factors predominantly contain the content. The general, main and sub-factors are described in Table 3 and in the following text, illustrated with quotes. Additional quotes are displayed in Supplementary Material Table 4. In the following text of the results section we will use the term “all groups of stakeholders” when the results are based on interviews of all groups of stakeholders and the term “interviewees” when they are based on some of the stakeholder groups.

**Table 3:** Overview of (groups of) stakeholders with general, main and sub-factors

STAKEHOLDER GROUP	GENERAL FACTORS	MAIN FACTORS	SUB-FACTORS
**RESIDENT**	**CHARACTERISTICS**	**PERSON**	Unlike other residents
		**BEHAVIOR**	Nature of the behavior
			Course of the behavior
			Severity of the behavior
			Unpredictability of the behavior
			Unclear triggers of the behavior
			Behavior considered as (partly) on purpose
			Behavior differs from personality before diagnosis of dementia
	**INTERACTION ISSUES WITH OTHER (GROUPS OF) STAKEHOLDERS**	**WITH OTHER RESIDENTS**	The resident’s behavior causes inconveniences and danger for the other residents Reactions of other residents negatively affect the resident’s behavior
		**WITH NURSING HOME STAFF**	The resident not understanding verbal requests
			The resident giving short answers/minimal reaction
			The resident not wishing to/not making any contact
			Inability of nursing home staff to read the resident’s emotions
			Nursing home staff not understanding the resident’s behavior and having no control over the behavior
		**WITH CARE STAFF SPECIFICALLY**	Not noticing signs of escalation of the resident’s behavior in a timely manner
			Positive moments with the resident are scarce
			Paying attention to the resident takes a lot of time
			Undertaking pleasant activities with the resident is problematic
			Applying compulsory treatment is difficult
**RELATIVE**	**CHARACTERISTICS**	**PERCEPTIONS**	Having a different perception of the behavior, treatment and care
			Finding it hard to accept that usual care could not always be provided
	**INTERACTION ISSUES WITH OTHER (GROUPS OF) STAKEHOLDERS**	**WITH NURSING HOME STAFF**	Nursing home staff insufficiently informs/involves relatives
			Relative has limited trust in (certain) care staff members
			Relative criticizes actions of care staff
			Relative crosses personal boundaries of care staff members
			Relative is ambivalent/uncommunicative about emotions and wishes for treatment
**CARE STAFF**	**CHARACTERISTICS**	**PERSONALITY ISSUES**	Different approaches and interactions with the resident due to different personalities of care team members
		**SKILLS ISSUES**	Having insufficient knowledge and experience
			Reports are of an insufficient quality
			Reflects insufficiently on own actions and feelings
		**ATTITUDE ISSUES**	Having a wait-and-see attitude/refraining from taking the initiative
			Not asking for help/asking for help too late
			Refraining from complying with the behavioral management approach that was agreed on
			Having a fatalistic attitude
			Differences in views on the behavior, approaches in dealing with the resident’s extreme challenging behavior and experiences of the behavior due to a difference in working shifts (day/night) and number of working hours
			Difference in opinions about appropriate care
			Difference in the extent to which the resident’s behavior is accepted
	**INTERACTION ISSUES WITHIN STAKEHOLDER GROUP**	**WITHIN CARE STAFF**	Little opportunity for formal and informal exchange of information
			Giving each other feedback is difficult
			New ideas from care staff members often receive a negative response from other care staff members
			Communication takes place indirectly
**TREATMENT STAFF**	**CHARACTERISTICS**	**BEING AT BAY**	Missing the whole picture of the situation and the resident’s behavior
			Only present during office hours
		**TREATMENT ISSUES**	Difficult to develop and implement a treatment plan
			Treatment plans have no effect/temporary effect
			The situation often needed to end as soon as possible
			Difficulties with prescribing medication
		**SKILLS ISSUES**	Having insufficient knowledge and experience
			Making treatment plans which are outdated/ impractical/unachievable/not feasible
			Unable to detect the needs of the care staff, meet their expectations or support them properly
			Involving external expertise too late
		**ATTITUDE ISSUES**	Being indecisive/taking little responsibility
			Undertaking too few actions
			Not informing themselves properly about the (severity of) the behavior
			Unaware of the expertise of care staff
	**INTERACTION ISSUES WITHIN STAKEHOLDER GROUP**	**WITHIN TREATMENT STAFF**	Different perceptions as to everyone’s responsibilities pertaining to the situation
			Not enough formal and informal exchange of information between the psychologist and elderly care physician
**NURSING HOME STAFF**	**INTERACTION ISSUES WITHIN STAKEHOLDER GROUP**	**QUALITY OF INTERDISCIPLINARY COMMUNICATION**	Limited exchange of information due to few meetings
			No room for reflection
			No room for giving each other feedback
			No room for an extensive analysis of the behavior
			Care staff members not communicating their needs, wishes and actions taken with the treatment staff
			Care staff members share incomplete and unclear information
			Treatment staff members insufficiently involving care staff in their plans
			Care and treatment staff not taking each other seriously or not listening to each other’s ideas/rationalizations for approaching the problem
		**INEFFICIENT WORK PROCESSES**	Indirect communication between care and treatment staff Inefficient communication due to a missing working agreement
**ORGANIZATION**	**CHARACTERISTICS**	**STAFFING ISSUES**	Short staffing and staff-turnover
			Excessive workload
		**UNIT**	Size of the unit
		**ORGANIZATIONAL NORMS**	Acceptance of the behavior by considering it as part of the dementia or the resident’s personality
		**ROLE OF MANAGEMENT**	Management staff insufficiently investing in solutions to improve the situation for the resident
			Management staff making decisions interfering with the clinical situation

### Resident

#### Characteristics

According to interviewees, it was challenging that the resident was unlike the other residents. This was mainly related to the resident being physically stronger and less cognitively impaired.

*“He (the resident) is just completely unlike all of the other residents we have. Which almost makes my stomach ache. That I think, imagine that we have placed that man (the resident) in a psychogeriatric ward while he is not as demented as everybody thinks he is.”* (Case 3, Unit manager in focus group discussion)

Furthermore, the resident’s behavior was considered highly complex and particularly challenging, due to its nature (e.g. aggression), course (constantly present or varying in frequency), severity, unpredictability and triggers remaining unclear. Sometimes the behavior was considered as (partly) on purpose, which lowered its acceptability. In other cases, interviewees reported that the resident’s behavior differed greatly from their personality before the diagnosis of dementia, and that it was therefore difficult for them to understand the behavior.

*“But what I noticed was that it was very taxing on the care team. That when she (the resident) pulled a care team member away with her, continually asked for their attention, that if the care team had to do something that required their focus, like distributing medication, yes, then it is impossible with her (the resident) standing beside you like that.”* (Case 4, Psychologist in individual interview)

*“It (the behavior) is just very fickle... That is what makes it so difficult.”* (Case 7, Care staff member in individual interview)

*“It (the behavior) is like a peat fire, so it arises somewhere and you don’t know where and when it will arise, or how fierce it will be when it arises.”* (Case 4, Elderly care physician in individual interview)

#### Interaction issues with other (groups of) stakeholders

The resident’s interaction with other residents was considered problematic by the interviewees; the resident’s behavior caused inconveniences and dangerous situations for the other residents and the responses of other residents triggered the resident’s behavior.

*“Also the stimuli that she (resident) receives from other residents...I feel that she should be in a low-stimulus environment... And of course she is here with other residents who also do all sorts of things... which end up being an extra trigger, so to speak.”* (Case 2, Care staff member in individual interview)

The interaction of the resident with the nursing home staff was also considered challenging, which was mostly attributed to the resident experiencing communication difficulties, delusions, diminished hearing or medication side-effects. The resident was often unable to understand verbal requests or express themselves when communicating with the nursing home staff, which contributed to the appearance of extreme challenging behavior and in experiencing the situation as an impasse. Occasionally the resident responded to nursing home staff with only a short answer or a minimal reaction and sometimes the resident did not make, or did not wish to make, any contact with them.

*“He (the resident) literally stands very close to you, with a story you could not make heads or tails of, you know, so that makes him angry too, or he pinches you or pushes you away, but also that look in his eyes, he doesn’t see you anymore.”* (Case 6, Unit manager in focus group discussion)

Furthermore, in some cases, nursing home staff were not able to make contact with the resident or understand the resident’s emotions, making it difficult for them to understand and have a grip on the behavior.

*“If you cannot get a hold on it (the behavior), that is what I find difficult. When I believe that I have tried everything and the behavior remains. Then you feel like your back is up against the wall.”* (Case 7, Certified primary nurse assistant in individual interview)

Several difficulties regarding the interaction of the resident with the care staff specifically were reported by nursing home staff and relatives. In three cases, care staff members did not notice signs of escalation of the extreme challenging behavior in time, leading to outbursts. Due to the extreme challenging behavior, caring for the resident could be very intense, contained scarce positive moments and required close attention and time, sometimes at the expense of the other residents.

*“When you are very busy with her (the resident), the other residents, say, pale in significance sometimes, because you constantly focus on her (the resident), by making sure that she remains calm. While there are another six people there who need attention too.”* (Case 2, Care staff member in focus group discussion)

In most cases, it was difficult to engage in pleasant activities with the resident and to provide care without the behavior occurring. In three cases, compulsory treatment was applied, such as putting the resident in a jumpsuit. This resulted in a heavy burden for the care staff members; the application of compulsory treatment conflicted with their norms and values, but it was considered necessary to ensure safety.

*“He did wear a jumpsuit for a while because he had smeared feces on himself. Well, he thought it was horrible to put the thing on and it was a hopeless job to get it on. Then I think, who are we doing this for? Since it is a disaster to get it off again. You trigger him even more then, yes, what exactly is this all for?”* (Case 3, Certified primary nurse assistant in individual interview)

### Relative

#### Characteristics

According to nursing home staff, relatives often had a different perception of the resident’s behavior and the required treatment and care. Nursing home staff of three cases said that relatives were not aware of the severity of the behavior or trivialized it. Furthermore, it was difficult for the relatives to accept that usual care could not always be provided due to the resident’s resistance to care and the severity of the behavior.

*“Things were sometimes played down too (by the relative), perhaps out of self-preservation, I always used to think, like when you were told that he (the resident) had been very aggressive. But, oh, never mind, fortunately you are all thick-skinned or it wasn’t too bad.”* (Case 1, Care staff member in individual interview)

#### Interaction issues with other (groups of) stakeholders

Both nursing home staff and relatives mentioned that care and treatment staff informed and involved relatives insufficiently, which led to dissatisfaction.

*“That family conversation revealed that we were not as well informed about certain issues, that something had gone wrong in the communication.”* (Case 7, Relative in individual interview)

On occasions, nursing home staff expressed the feeling that they could not do anything right as relatives had limited trust in them. Interviewees said that relatives crossed personal boundaries of care staff by demanding specific care activities, such as dressing the resident despite the resident’s resistance to care. This led care staff to feel that the relatives did not acknowledge the resident’s problematic behavior. Moreover, several relatives were ambivalent or uncommunicative about their emotions and wishes for treatment. Therefore, it was difficult for nursing home staff to gain support for their care and treatment plans, which sometimes led to a delay in executing the planned care.

*“ You thought, okay, she (the relative) understands, she got my message and, when the family conversation was finished or maybe half a day later, she (the relative) said something completely different... The fact that she (the relative) wasn’t always consistent, that also made it difficult to get her to support the multidisciplinary team and support the agreements.”* (Case 1, Psychologist in individual interview)

### Care staff

#### Characteristics

Interviewees experienced that the variety of characteristics and personalities of care staff resulted in different approaches with the resident. For example, a care staff member who was male or perceived as busy could trigger the resident’s behavior. In one case this meant that only a limited number of care staff members were able to prevent behavioral outbursts from the resident.

*“In any case, it has to do with whether people have a certain calm. For instance, care staff members who tend to come down on residents if they resist. Well, if you have done that to him (the resident) once, you are forever in his bad books.”* (Case 3, Elderly care physician in individual interview)

Furthermore, skills issues of care staff emerged as another sub-factor in experiencing the situation as an impasse. All groups of stakeholders highlighted that the care staff had insufficient skills and knowledge of extreme behavior.

*“And sometimes I think that we were not trained to support and supervise this gentlemen (the resident) in the progress of his illness. Just where knowledge is concerned. This is one of these extreme cases.”* (Case 3, Care staff member in individual interview)

Another commonly mentioned difficulty concerned reporting by care staff. It was noted that care staff members often did not report the challenging behavior or that reports were of an insufficient quality. This was attributed to reporting not being possible or time consuming, the resident’s behavior being considered ‘usual’, difficulty in expressing the severity of the behavior in words and not wanting to upset the relatives as they had access to the digital reports. Due to these issues regarding reporting, interviewees felt the severity of the behavior was registered insufficiently and was therefore presented to the treatment staff much too late.

*“But at night, you feel like, well it is so busy now and it has been for weeks that I have to rush off to the next bell in a moment, I will write it later and there comes a time you don’t write it at all.”* (Case 5, Care staff member on the nightshift in individual interview)

*“It is difficult to report, like, I was hit and backed into a corner, because you know that family will read that too... then you feel like you want to play it down.”* (Case 1, Unit manager in individual interview)

Interviewees including care staff members of six cases mentioned that care staff rarely reflected on their own actions and feelings regarding the resident’s behavior, due to a lack of skills or time*.*

*“Don’t assume that somebody else (other care staff members) might not know or does not have the relevant knowledge and so just really ask, like, how do you experience it and what do you run up against and what do you feel is difficult with this?”* (Case 3, Certified primary nurse assistant in individual interview)

*“To influence the behavior yourself, then I think there is a lot to win in that respect, that you have to critically assess, like, how do you deal with that behavior, what is my own role in that.”* (Case 7, Elderly care physician about the care staff in individual interview)

Finally, several care staff attitude issues played a role in experiencing the situation as an impasse. In the majority of cases, interviewees perceived that several care staff members had a wait-and-see attitude and they refrained from taking their role or from complying with the behavioral management approach that was agreed upon. Interviewees noted that care staff members found it difficult to ask for help, partly due to a fear of failure, meaning that they sometimes did not ask or asked much too late. Care staff reported that they felt alone and that their voice was not heard by the treatment staff*.*

*“I do find it difficult to say to an elderly care physician or a psychologist, like, listen, we all find this difficult, could you provide us with a little more guidance? It feels like there is a threshold you are crossing.”* (Case 1, Certified primary nurse assistant and care staff member in focus group discussion)

*“I: What would you need? From the elderly care physician, from the psychologist?’*

*Well, that they hear and listen to us... that your opinion is also heard.”* (Case 3, Care staff member in individual interview)

From the interviewees’ perspective, care staff members often had a fatalistic attitude towards the resident and held negative views about their behavior. As a result, it required greater effort for them to care for the resident and perform certain job tasks, such as reading reports about the resident’s behavior.

*“At a certain moment you become prejudiced, you enter the room (of the resident) with apprehension, you have to dig really deep to find empathy... Because at a certain moment you already have the feeling, like, I do not want to help him (the resident) anymore, for it always ends up being wrong anyway. And that is not fair to him, since he doesn’t have a fair chance that way.”* (Case 7, Care staff member in individual interview)

Not all of the care staff members experienced the situation as an impasse, which seemed to be related to a difference in working shifts (e.g., day versus evening shift) and in the number of working hours. Moreover, care staff member’s views, approaches and experiences with the challenging behavior differed. For example, a certified primary nurse assistant said that she did not pick up on the signals highlighted by other care staff members about the severity of the behavior, which led to a delay in involving the treatment staff. According to the interviewees, various views were expressed about the appropriate care among care staff members which led to different approaches with the resident. For instance, some reassured a particular resident by crawling into bed with them, whereas others would not. Furthermore, care staff members differed in the extent to which they accepted the resident’s behavior; sometimes they let their personal boundaries be crossed.

*“Everyone has their limits, of course, and with some people the limit is this and with others the limit is that and I think that some have gone on longer than was good for them.”* (Case 2, Care staff member in focus group discussion about care team members in general)

#### Interaction issues within stakeholder group

Interviewees noted that too few care team meetings were held and that in these meetings relevant topics, such as how to deal with the behavior, were often not discussed. As a consequence, several care staff members had insufficient insights into the behaviors and how to address them. Furthermore, giving each other feedback about one’s actions was considered difficult, as care staff members were quickly offended and avoided confrontation. New ideas often received a negative response from other care staff members.

*“Because sometimes you leave after certain situations that someone went through with that gentleman (the resident), you go home, you’re still completely full of emotions or with feelings that you didn’t even have at the time during work. And are unable to just share it with each other, what happened now, what did that do to you?” (*Case 6, Nurse in focus group discussion)

Interviewees felt that there was a lack of dialogue between the care staff members about different attitudes, experiences and views regarding the situation due to indirect communication. Communication was further hampered between care staff members on different shifts.

*“There’s a powerlessness that I can’t explain things properly to the night shift care staff or that it doesn’t get through to them (night shift care staff) as to why we are not using medication right now. I often felt that I had to defend Ms … (name resident) to the night shift care staff.”* (Case 5, Certified primary nurse assistant in individual interview)

### Treatment staff (including unit manager)

#### Characteristics

To begin with, treatment staff members were said to miss the whole situation as they were only present during office hours and therefore at bay. They themselves mentioned to experience difficulties in treating the resident optimally. The complexity of the resident’s behavior slowed the development and implementation of a treatment plan that often also did not work, or only worked temporarily. The severity of the behavior and its consequences for the other residents and nursing home staff often required a swift resolution, which prohibited an extensive analysis of the behavior. Moreover, difficulties with medication prescribed were reported. Medication was frequently prescribed instantly, which interfered with the developed treatment plan. Finding appropriate medication was difficult given the phasing out, side-effects and delicate balance between under- and over-sedation.

*“And then you reach the point of yet trying another medication with consequences, she (the resident) becomes very drowsy again, she starts falling more, she doesn’t get involved in the home community anymore, yes, you don’t want that either, you want to be able to give her a dignified existence too.”* (Case 7, Unit manager in individual interview)

Two other main factors in experiencing the situation as an impasse concerned skills issues and attitude issues of the treatment staff. Interviewees similarly believed that treatment staff members had insufficient knowledge of and experience with extreme behavior. Treatment staff members made treatment plans which were outdated, impractical, unachievable and/or unfeasible in four cases. Furthermore, interviewees experienced that treatment staff members were unable to detect the needs of care staff members, meet their expectations or support them properly. In addition, treatment staff members insufficiently informed the care staff. All groups of stakeholders mentioned that, in retrospect, treatment staff repeatedly tried several interventions and involved external expertise, such as a geriatric psychiatrist or the CCE, only at the very last moment. Moreover, interviewees said that treatment staff members were indecisive and took little responsibility for the situation, undertook too few actions and did not inform themselves properly about the situation by visiting the unit or talking to care staff members. Finally, they were unaware of the care staff’s expertise.

*“It took me quite a while to see the seriousness of the problem. That is my personal opinion, at least. That afterwards I say, like: maybe I should have been a little more on top of it at the start.”* (Case 3, Elderly care physician in focus group discussion)

*“I think sometimes they don’t realize enough just how much expertise the care staff already has and what they all did before they (the psychologist and elderly care physician in training) arrived.”* (Case 1, Unit manager in individual interview)

#### Interaction issues within stakeholder group

Treatment staff members indicated that they had different perceptions as to everyone’s responsibilities pertaining to the situation and that there was not enough formal and informal exchange of information between the psychologist and elderly care physician.

*“And in addition, I’ve found it difficult to really find a team feeling with him (the elderly care physician), I’ve felt like a lot was done individually despite initiatives to do more together.”* (Case 7, Psychologist in individual interview)

### Nursing home staff

#### Interaction issues within stakeholder group

According to all groups of stakeholders, a prominent main factor regarding the interaction within the nursing staff concerned the interdisciplinary communication. Several issues were similar to those within the care staff and treatment staff, such as the limited exchange of information due to few meetings, little time for reflection, giving each other feedback or performing an extensive analysis of the behavior. Additional issues concerned care and treatment staff not involving each other beyond those meetings. From the interviewees’ perspectives, care staff members did not communicate their needs, wishes and actions taken with the treatment staff. The scarce information they did share was incomplete and unclear as it was difficult for them to express the severity of the behavior.

*“We (care staff) didn’t show enough that we needed help. We thought it would be fine.”* (Case 3, Care staff member in individual interview)

*“But in the beginning, I actually didn’t get any signals from the care staff that they had a problem with it... I think they share it mainly with each other and maybe don’t even make it very clear to the psychologist just how bad it is.”* (Case 3, Psychologist in individual interview)

Contrastingly, it was felt that treatment staff members insufficiently involved care staff in their plans.

*“A while back there was also something to do with the gentleman (the resident), I was on duty that day but I was not asked about it. Then it seemed like the elderly care physician, the psychologist, my unit manager and the quality nurse sat down and decided for us.”* (Case 3, Care staff member in individual interview)

Furthermore, interviewees felt that care and treatment staff did not take each other seriously or did not listen to each other’s ideas and rationalizations when approaching the problem.

*“The care staff also felt that they were not taken seriously and what they were very often told by the elderly care physician was ‘Yes I don’t have any miracle pills’, but that is not the question, we are asking for him to help us, pay attention, listen, shadow us for a moment... help us carry the load.”* (Case 6, Unit manager in individual interview)

*“We (care staff) did have some frustration as a team and also personally. He (the elderly care physician) still doesn’t consider it a crisis, while we’ve had concerns about that for a year with Ms … (name resident)... by that point, we actually felt disrespected. “* (Case 2, Care staff member in individual interview)

A second main factor concerned inefficient work processes. Similar to the situation among care staff, indirect communication between care and treatment staff was an issue. Face-to-face discussions often did not take place. For example, communication occurred through an intermediary, such as a nurse, as care staff members were not allowed to contact the elderly care physician without involving an intermediary.

*“Suppose there is an escalation and we need the elderly care physician at that moment, then there is a nursing station in between, so we actually have to call them first before an elderly care physician comes...That is yet another threshold you have to cross. Basically, we feel that the nurse does not know the resident, but we do.”* (Case 1, Nurse in focus group discussion)

Moreover, interviewees said that communication was inefficient due to a lack of a working agreement on how to contact each other. Occasionally, care staff members shared information about the resident with the psychologist and elderly care physician on separate occasions, with differing information. It was difficult for treatment staff to get a clear and complete picture about the behavior because they mostly spoke with only one care staff member, which was usually the same person, every time (commonly the certified primary nurse assistant) or rather, with a different care staff member each time. Moreover, there were occasions when they did not speak with the correct person (e.g., a trainee care staff member).

*“When you come, you talk to one care staff member and the next time you talk to another care staff member and they just have a slightly different opinion or a different perception or a different feeling... you then assume that such a care staff speaks with one voice, that’s quite difficult.”* (Case 4, Elderly care physician in focus group discussion)

#### Consequences of interaction issues within nursing home staff

Due to the abovementioned issues regarding communication and work processes among nursing home staff, experiences with and views on the resident’s behavior differed, which could lead to disagreements about the developed plans. In dealing with the behavior, feelings of powerlessness and failure prevailed. However, a number of staff members did not give up hope and continued with their search for a solution to manage the extreme behavior. Others gave up hope and resigned themselves to the situation, which sometimes even led to care staff members accepting that they were physically injured by the resident. Both of these coping mechanisms led to prolonged decision-making processes.

*“You have hope that it (the behavior) will get better. At some point you think that maybe it’s because of a certain reason, or that it’s an incident, after some time you think well maybe it (the behavior) will stay like this.”* (Case 1, Elderly physician in training in individual interview)

*“Yes, at some point you shut up about it too, yes, let’s all just do it. And I do think that this has happened. That we all think, well let’s just do it, because we won’t manage it (the behavior) anyway.”* (Case 4, Certified primary nurse assistant in individual interview)

### Organization

#### Characteristics

Staffing issues was mentioned by interviewees as one of the difficulties in experiencing the situation as an impasse. All participating units were short-staffed and their staff turnover was a barrier for optimal resident care. Moreover, care staff members highlighted not having enough time for particular residents or others due to an excessive workload.

*“And occasionally you’ll just ignore her (the resident), because then the workload is such that you think, well, I have to go to the others (other residents) first... that you don’t actually have enough time to sit quietly with her.” (Case 5, Care staff member in individual interview)*

In all cases, the size of the resident’s unit was mentioned as a problem by the interviewees. Six residents lived on a small-scale unit where one care staff member had to divide their attention across them. Care staff members did not receive a clear and complete overview of the resident’s behavior. In one case, the resident lived on a large-scale unit and was therefore easily triggered by a variety of stimuli stemming from the other residents.

*“She (the resident) cannot be attended to 24 hours a day, she also walks around the unit and sometimes there is one care staff member who has six or seven other residents. That care staff member is not standing there all the time checking what she (the resident) is doing.”* (Case 4, Elderly care physician in individual interview)

Furthermore, organizational norms and values in five of the seven cases led to acceptance of the behavior by considering the extreme behavior as part of the dementia or the resident’s personality, rendering it more acceptable.

*“It has become part of the culture though, the idea that we think that it (the behavior) is becoming normal.”* (Case 4, Care staff member in individual interview)

*“You notice that they (the care staff) often put up with things and think things are normal for quite a long time. Under the guise of, well, that’s just part of the pathology and you can’t blame him (the resident). But they are still being beaten and pinched.”* (Case 1, Psychologist in individual interview)

Finally, interviewees described the role of the management of the nursing home as a main difficult factor. The interviewees reported that the management staff of the nursing home insufficiently invested in solutions to improve the situation regarding residents with extreme behavior, such as making funds available to invest in environmental adjustments to influence the resident’s behavior. In one case, the management staff made decisions which interfered with the clinical situation.

*“And that has more to do with the fact that management has started to interfere with the content of the case, which really does not please me.”* (Case 6, Elderly care physician in individual interview)

## Discussion

This is the first study in which an in-depth exploration of situations of extreme challenging behavior concerning nursing home residents with dementia was conducted. We found that several characteristics and attitudes of nursing home staff, as well as their interactions, contributed to their experience of the situation as an impasse. In particular, the resident’s characteristics, together with suboptimal mono- and interdisciplinary communication and collaboration were experienced as the greatest difficulties. Nursing home staff members kept searching for a solution to manage the resident’s extreme challenging behavior or lost hope. In the end, they did not know how to cope with the situation any longer and consulted external expertise.

Part of our findings are in line with earlier studies such as the difficulties experienced by the nursing home staff with the nature, extremity and persistency of the behavior, developing a clear treatment plan and prescribing medication [11, 30]. Moreover, the disparity in views and attitudes of the staff and their need for more knowledge is a familiar theme across nursing home care [30–33]. A conflicting result with our study concerns a review in which a positive influence of small-scale units on the residents’ behavior was described [34], compared to the negative influence found in our study. It is possible that small-scale units can contribute to a general reduction of challenging behavior, but are not suitable for residents exhibiting extreme behaviors. It is also possible that the limited use of mono- and interdisciplinary meetings on these units is also a factor, which play a greater role for residents with extreme behaviors.

Several of our findings are connected to the nursing home staff’s professional attitude. It appeared to be difficult for staff members to reflect on their own and others’ behavior, which was further complicated by the circumstances, such as the lack of interdisciplinary meetings. As well as this, care staff members experienced problems with reporting the resident’s behavior in a structured, objective and detailed manner. They were afraid to upset the relatives with the severity of the situation, as relatives have access to the (digital) resident files. This appears to be specific to Dutch nursing home organizations, who promote and facilitate this. Furthermore, setting personal boundaries towards, not only the resident, but also their relatives, was a challenge for care staff members. Striking a balance between delivery of personal care while maintaining boundaries has been found to be difficult for many nursing assistants [35]. Indeed, an optimal balance between personal intimacy and maintaining a professional attitude is lacking in the literature on person-centred care, although the nursing literature stresses the importance of boundaries and a good balance between distance and involvement [36, 37]. Too little attention on this balance could result in person-centred care being misinterpreted by care staff members and may lead to a serving attitude and culture of over-acceptance of challenging behavior as ‘part of the job’ [38], ultimately compromising the wellbeing of nursing staff [37, 39]. Probably suboptimal professional behavior of nursing home staff is less relevant in cases with less severe challenging behavior and is especially required in residents with extreme challenging behavior. Namely, coping with extreme challenging behavior may engender a need for more personal leadership and insight into one’s own behavior [40, 41]. It is not just about having the necessary knowledge and experience, but also about the way of dealing with tasks, oneself and others within the broader situation. Although in current Bachelor training programs for nurses in the Netherlands, professionality is included as one of the required competences [42], in training programs for vocationally trained registered nurses, this is not the case. The newly developed ambassador trajectory for certified nurse assistants and the introduction of nurses with a Bachelors education in the nursing home may contribute to nursing leadership and empower members of the care team [43].

Our study also showed that treatment staff members did not recognize the knowledge and expertise of care staff, were unable to detect their needs or to support them properly. An earlier study suggested that acknowledgement of nurses’ competencies by physicians is one of the keys to improving interdisciplinary collaboration [44]. Especially in the case of residents with extreme challenging behavior, treatment staff members should create an environment in which care staff members are sufficiently supported and their professionality is appreciated.

### Clinical implications

In sum, the findings of our study clarify that situations of extreme challenging behavior are experienced as an impasse by nursing home staff and relatives due to the resident’s specific characteristics together with problems regarding mono- and interdisciplinary communication and collaboration of nursing home staff. As we now know these important contributing factors, we could develop interventions based on knowledge about mono- and interdisciplinary communication and collaboration to prevent these impasses in the future. We think that particularly in these complex cases, communicating and collaborating intensively is the key to managing these situations and finding the most optimal approach. Teaching nursing home staff members solid communicative and reflective skills, tackling learned helplessness and developing self-awareness are important aspects to take into account [45, 46]. Furthermore, having attention for each other and supporting each other in these complex cases, besides bearing the responsibility together, could be helpful for nursing home staff as it would make it a less heavy burden to bear. Sufficient time needs to be available for regular meetings in which there are opportunities and a safe atmosphere to share views, give feedback and inform each other about the interventions and treatment plans. In addition, working uniformly and methodically seems to be very important and requires further attention, especially as it was found to be effective in reducing challenging behavior, but difficult to implement [47, 48]. This mainly concerns performing a good assessment and evaluation of treatment plans and medication. Also, the use of structured ways of communication, for instance a communicative framework based on the SBAR (Situation, Background, Assessment, and Recommendation) approach may be helpful [49].

### Strengths and limitations

We performed a high quality, in-depth exploration of experiences with extreme challenging behavior involving all relevant stakeholders, using a combination of methods for data collection (data triangulation) and analytical techniques (analysis triangulation), having multiple researchers involved (investigator triangulation) and organized (consensus) meetings with each other and all authors. This approach enhanced the reliability and trustworthiness of the results [20, 21]. Though, it is important to realize that our study focuses on extreme situations, all cases concerned impasses for which external expertise was requested. The issues uncovered in this study probably result in less severe problems when presented in less severe situations. In addition, only the experiences of nursing home staff and relatives were reported in this study. Therefore, inferences about extreme challenging behavior in general cannot be made. Furthermore, the characteristics and roles of the researchers could have influenced the analysis of the data [50]. Moreover, to ensure a safe environment, we did not share all the information obtained from the individual interviews in the focus group discussions and although we noticed in the individual interviews that interviewees had certain views about each other, we did not investigate how these were related. Both of these factors could have provided additional insights. Finally, external validity of the results is unclear as these reflect the Dutch cultural, societal, and health care contexts.

## Conclusion

Situations of extreme challenging behavior in nursing home residents with dementia can be experienced as an impasse by nursing home staff and relatives, especially due to the resident’s characteristics together with suboptimal mono- and interdisciplinary communication and collaboration of nursing home staff. Although the conditions for high-quality care are present in the nursing home, namely the wide range of expertise and committed relatives, suboptimal collaboration and insufficient work processes still exist. The contributing factors found in this study provide important insights into the complexity and extent of these situations and offer caregivers targets to improve the provided care, treatment and interdisciplinary collaboration for nursing home residents with dementia and extreme challenging behavior. Situations of extreme challenging behavior require specific skills due to their complexity. Collaborating intensively, working methodically and achieving the right balance between personal intimacy and a professional attitude are key to dealing with those situations. Moreover, involving external expertise at an earlier point in time and finding the most optimal solution, which may be to transfer the resident to a specialized care unit, are important. To obtain further insight into situations of extreme challenging behavior, future research should investigate the added value of the use of assessment instruments to measure the frequency, severity and impact of the behavior, focus on the quality of (digital) reports and explore if a more structured, objective and detailed way of reporting could assist care staff members.

## Supplementary Information

**Additional file 1:.** Supplementary material Table 1. Detailed applied methodology following the consolidated criteria for reporting qualitative studies (COREQ) 32-item checklist.^1^

**Additional file 2: Supplementary material Table** **2****.** Topic list for semi-structured in-depth interview with interviewees.

**Additional file 3: Supplementary material Table** **3****.** Topic list for focus group discussions with interviewees.

**Additional file 4: Supplementary Material Table 4.** Additional quotes for all described general, main and sub-factors of the six (groups of) stakeholders.

## Data Availability

The datasets generated and/or analysed during the current study are not publicly available as interviewees have not given their permission for data sharing outside of the research group. Further information is available from the corresponding author on reasonable request.
