# Supplementary material for: Losing hope or keep searching for a golden solution: an in-depth exploration of experiences with extreme challenging behavior in nursing home residents with dementia
Source: BMC Geriatr. 2022 Sep 16;22:758. doi: 10.1186/s12877-022-03438-0 (PMC9479311; doi:10.1186/s12877-022-03438-0)
Supplement: Supplementary file 1 — Additional file 1:. Supplementary material Table 1. Detailed applied methodology following the consolidated criteria for reporting qualitative studies (COREQ) 32-item checklist.1 [file 12877_2022_3438_MOESM1_ESM.docx]

**Supplementary material Table 1. Detailed applied methodology following the consolidated**

**criteria for reporting qualitative studies (COREQ) 32-item checklist.^1^**

| **No. Item** | **Guiding questions/description** | **Application** |
| --- | --- | --- |
| **Domain 1: Research team and reﬂexivity** | |  |
| *Personal Characteristics* | |  |
| 1.Interviewer/facilitator | Which authors conducted the interview? | AV conducted the individual interviews,  AP and DG moderated the focus group discussions. AV asked additional questions, and observed body language and interactions between the interviewees. |
| 2. Credentials | What were the researcher’s credentials? | MD |
| 3. Occupation | What was their occupation at the time of the study? | Elderly care physician in training and PhD student.^2^ |
| 4. Gender | Was the researcher male or female? | Female |
| 5. Experience and training | What experience or training did the researcher have? | AV: medicine, entry-level course in Atlas.ti, basic course qualitative health research.  Research team: medical (SZ, MS, RK), psychological (DG), nursing (science) (AP) and all are specialized in elderly care. |
| *Relationship with participants* | |  |
| 6. Relationship established | Was a relationship established prior to study commencement? | AV was not acquainted with interviewees before the interview. |
| 7. Participant knowledge of the interviewer | What did the participants know about the researcher? e.g. personal goals, reasons for doing the research | A briefing letter about the purpose of the study and practical information about the interview was sent to the relatives of eligible candidates. This letter also mentioned that the interviewer was an elderly care physician in training (AV). |
| 8. Interviewer characteristics | What characteristics were reported about the interviewer/facilitator? e.g. bias, assumptions, reasons and interests in the research topic | The occupation of the interviewer was given in the briefing letter. |
| **Domain 2: Study design** | |  |
| *Theoretical framework* | |  |
| 9. Methodological orientation and theory | What methodological orientation was stated to underpin the study? e.g. grounded theory, discourse analysis, ethnography, phenomenology, content analysis | Thematic analysis was used (both inductive and deductive), including conventional content analysis.^3-5^ |
| *Participant selection* |  |  |
| 10. Sampling | How were participants selected? e.g. purposive, convenience, consecutive, snowball | We used consecutive sampling to select cases. Cases were assessed for inclusion by two coordinators of the Centre for Consultation and Expertise (CCE)^6^ and by AV and DG by verifying the inclusion criteria: a) the resident had dementia and extreme challenging behavior which affected their quality of life according to the professionals who reported the case to the CCE; b) there was no obvious easily treatable cause for the challenging behavior; c) the behavior was experienced as very difficult to cope with by the involved nursing home staff and they had been unable to treat the challenging behavior satisfactorily; d) the challenging behavior consisted of aggression and/or vocally disruptive behavior and/or agitation; e) the resident had no acute life-threatening diseases; and f) they had been staying in the nursing home for at least 4 weeks. |
| 11. Method of approach | How were participants approached? e.g. face-to-face, telephone, mail, email | - The coordinator of the CCE telephoned the notifying party and asked them for the contact information of the elderly care physician of the resident. - The coordinator telephoned the elderly care physician and asked if AV was permitted to contact them. - AV contacted the elderly care physician within a week after notification and gave them information about the study. The elderly care physician was asked to talk with their manager regarding permission for participation in the study. Also, a briefing letter about the rationale and aim of the study was sent to them by post or by email. - When the elderly care physician and supervisor agreed to participate in the study, they were asked to sign a consent form. - The elderly care physician telephoned the relatives of the nursing home resident and asked if AV was permitted to contact them. - AV contacted the relative within a week and gave them information about the study. A briefing letter and a consent form were sent to them by post or by email. The relative was given two weeks to think about the participation. - The relative was contacted again by AV within two weeks by telephone or email to ask for consent. After agreement to participate in the study, relatives were asked to sign the consent form and appointments were scheduled for the interviews. - When a case was deemed appropriate for inclusion by the elderly care physician and the unit manager of the nursing home, intensively involved nursing staff members (as mentioned in Table 1) and the relative were asked for consent to participate in the study. For consent a written consent form was used. |
| 12. Sample size | How many participants were in the study? | Of the 19 applied cases, 9 were assessed as eligible according to the inclusion criteria. The other 10 cases were excluded, because a) they did not meet the inclusion criteria; b) the consultation question was too narrow; c) AV was absent due to an internship; or d) there was a sufficient number of cases at that time.  Individual interviews: 42 interviewees.  Focus group discussions: 52 interviewees. |
| 13. Non-participation | How many people refused to participate or dropped out, and why? | In 2 cases, the elderly care physician/ nursing home refrained from participating in the study. These cases were therefore not included in the study. |
| *Setting* |  |  |
| 14. Setting of data collection | Where was the data collected? e.g. home, clinic, workplace | The individual interviews with nursing home staff were held during April-December 2016 in the nursing home of the resident, while interviews with relatives took place during April-October 2016 at their own home (N=4) or in the nursing home (N=3).  The focus group discussions with nursing home staff were held during April 2016- January 2017 in the nursing home of the resident. |
| 15. Presence of non-participants | Was anyone else present besides the participants and researchers? | Interviewees had the opportunity to be interviewed together with a second representative of the same perspective. No non-participants were present during the interviews. |
| 16. Description of sample | What are the important characteristics of the sample? e.g. demographic data, date | See Table 2. |
| *Data collection* |  |  |
| 17. Interview guide | Were questions, prompts, or guides provided by the authors? Were they pilot tested? | For each group of stakeholders and for the focus group discussions, AV used a semi-structured interview guide, in which the main questions and sub-questions were displayed. These interview guides were also used as an extra check to make sure all topics were discussed. The interview guides were not pilot tested. Planned, informal and floating prompts were used during the interviews.  The topic list of the focus group discussions could change after each focus group discussion: debriefing took place between the moderator and observer to discuss the findings and to identify topics that could be explored further. |
| 18. Repeat interviews | Were repeat interviews carried out? If yes, how many? | No |
| 19. Audio/visual recording | Did the research use audio or visual recording to collect the data? | All interviews were audio-taped and transcribed verbatim by three medical students (LS, KE and LB) and a professional transcription office (6), eliminating any names or privacy-related information. The transcripts were read closely and cross-checked against the tapes for accuracy by AV. |
| 20. Field notes | Were ﬁeld notes made during and/or after the interview or focus group? | Field notes were made by AV during and after each individual and focus group discussion. |
| 21. Duration | What was the duration of the interviews? | The individual interviews lasted between 60 and 90 minutes.  The focus group discussions lasted between 120 and 150 minutes. |
| 22. Data saturation | Was data saturation discussed? | All authors discussed findings and data saturation after each case. We expected to include ten cases, but stopped inclusion after interviewing for seven as we had reached data saturation, determined by all authors. For the seventh case, no new codes were added to the coding tree. ^7^ |
| 23. Transcripts returned | Were transcripts returned to participants for comment and/or correction? | A summary of each transcription was made (AV) and returned to the interviewees for checking. After a few adjustments based on comments from eleven interviewees, all agreed with the summaries. |
| **Domain 3: Analysis and ﬁndings** | |  |
| *Data analysis* |  |  |
| 24. Number of data coders | How many data coders coded the data? | 5: AV, AP, EV, MW and KM.  For the first case, 5 interviews were coded twice by two coders (AV and AP) and the coded transcripts were discussed in a consensus meeting. The other 2 interviews of the first case were coded by AV and checked by AP. After analysis of the first case, the coding tree was discussed in a meeting with AP, AV and DG, and also in a meeting with all authors. A modified version of this coding tree was used for the analysis of the other cases.  For the other cases, except for the fourth case, one interview was coded twice by two coders. The coded transcript of the twice coded interviews were discussed between these two coders, and as a result, further changes were made in the coding tree. The other interviews were coded by one person, with all codes checked by AV. If necessary, changes were made. After analysis of the last case, the latest version of the coding tree was used for re-coding of the transcripts of the other six cases to improve analysis accuracy (MW). |
| 25. Description of the coding tree | Did authors provide a description of the coding tree? | No, but this is available from the authors on request. |
| 26. Derivation of themes | Were themes identiﬁed in advance or derived from the data? | For each case, consensus meetings took place with the two data coders involved (AV, AP, EV, MW, KM) and one of the authors (DG). In these meetings, the case was discussed, categories were refined into definitive themes and sub-themes and an overall theme was defined. Per case, mind maps were made for within-case analysis (EV, AV).The mind maps were discussed in meetings with all authors. For a cross-case analysis, one mind map was made consisting of all mind maps together (AV). A final graphic representation of the themes, sub-themes and their connections was made after several group discussions.  Three main factors were derived from the data. |
| 27. Software | What software, if applicable, was used to manage the data? | Analysis with Atlas.ti version 7.1.4. was conducted during data collection (Atlas.ti Scientific Software Development, Berlin, Germany). |
| 28. Participant checking | Did participants provide feedback on the ﬁndings? | Eleven interviewees provided feedback on the summaries of their interview or focus group discussion. After a few adjustments, they agreed with the summaries. |
| *Reporting* |  |  |
| 29. Quotations presented | Were participant quotations presented to illustrate the themes/ﬁndings? Was each quotation identiﬁed? e.g. participant number | Yes, see Results section of the manuscript and Supplementary material table 4. Quotes were translated into English by translation agency Univertaal |
| 30. Data and ﬁndings consistent | Was there consistency between the data presented and the ﬁndings? | Yes |
| 31. Clarity of major themes | Were major themes clearly presented in the ﬁndings? | Yes, see Results section of the manuscript, Table 3 and Figure 1. |
| 32. Clarity of minor themes | Is there a description of diverse cases or discussion of minor themes? | Yes |

Abbreviations: AV, Annelies Veldwijk; SZ, Sytse Zuidema; MS, Martin Smalbrugge;

RK, Raymond Koopmans; DG, Debby Gerritsen; AP, Anke Persoon; LS, Lex van Son; KE,

Kyra Ekker; LB, Leonie Buijsse; EV, Erica de Vries; MW, Mandy Wijnen; KM, Kim Maassen.

1. Tong, A., Sainsbury, P., & Craig, J. (2007). Consolidated criteria for reporting qualitative research (COREQ): a 32-item checklist for interviews and focus groups. International journal for quality in health care, 19(6), 349-357.
2. Koopmans R, Pellegrom M, van der Geer ER. The Dutch Move Beyond the Concept of Nursing Home Physician Specialists. *J Am Med Dir Assoc* 2017;18(9):746-9.
3. Braun V, Clarke V. Using thematic analysis in psychology. *Qual Res Psychol* 2006;3(2):77-101.
4. Elo S, Kyngas H. The qualitative content analysis process. *J Adv Nurs* 2008;62(1):107-15.
5. Hsieh HF, Shannon SE. Three approaches to qualitative content analysis. *Qual Health Res* 2005;15(9):1277-88.
6. <https://www.cce.nl/english>
7. Evers JC, van Staa AL. Qualitative Research Methods Overview. In: Albert Mills GDEW, editor. Encyclopedia of case study research Part 2 Thousand Oaks: Sage Publications; 2010. p. 5.
