# Supplementary material for: Losing hope or keep searching for a golden solution: an in-depth exploration of experiences with extreme challenging behavior in nursing home residents with dementia
Source: BMC Geriatr. 2022 Sep 16;22:758. doi: 10.1186/s12877-022-03438-0 (PMC9479311; doi:10.1186/s12877-022-03438-0)
Supplement: Supplementary file 2 — Additional file 2: Supplementary material Table 2. Topic list for semi-structured in-depth interview with interviewees. [file 12877_2022_3438_MOESM2_ESM.docx]

**Supplementary material Table 2. Topic list for semi-structured in-depth interview with interviewees.**

| **Topic** | **Interview questions** | **Additional sub-questions** |
| --- | --- | --- |
| **Nature and course of challenging behavior** | What does the extreme challenging behavior of the resident consist of? | Can you provide examples?  How was the extreme challenging behavior expressed over time?  Was the resident’s extreme behavior already present before nursing home admission, and if yes, how did that manifest? (asked to relatives)  What are possible reasons for the behavior in your opinion?  Which factors influenced the resident’s extreme challenging behavior in your opinion?  Which role do you think that the environment plays in the resident’s extreme challenging behavior?  Could there be a connection between the resident’s extreme challenging behavior and prior events in the resident’s life? (asked to relatives) |
| **Actions undertaken** | Asked to professionals: Which action(s) did you undertake to change the situation regarding the resident’s extreme challenging behavior?  Asked to relatives:  Which action(s) were undertaken to change the situation regarding the resident’s extreme challenging behavior? (asked to relatives) | For what reasons did you undertake these action(s)?  What added value do you think that your actions had on the resident’s extreme challenging behavior?  What do you think about the actions undertaken by other professionals?  In retrospect, what would you have done differently?  What do you think about these actions?  What role did you play in these actions?  What added value do you think that your role had on the resident’s extreme challenging behavior?  In retrospect, what could have been done differently in your opinion? |
| **Factors contributing to an impasse** | Do you think that a certain point of crisis was reached? | If yes, which factors contributed to this point of crisis?  What difficulties did you experience when dealing with the resident’s extreme challenging behavior?  Why is the resident’s extreme challenging behavior untreatable/unsolvable? Do you have any ideas on that? (asked to professionals)  As a professional, do you think you have tried everything to solve the problem? (asked to professionals) |
| **Impact of the resident’s extreme challenging behavior** | What is the impact of the resident’s extreme challenging behavior on the environment? | What was the impact of the resident’s extreme challenging behavior on you up until now?  What is the impact of the extreme challenging behavior on the resident themselves?  What is the impact of the resident’s extreme challenging behavior on the other residents?  What is the impact of the resident’s extreme challenging behavior on relatives? (asked to professionals)  What is the impact of the resident’s extreme challenging behavior on treatment staff members?  What is the impact of the resident’s extreme challenging behavior on care staff members? |
