# Supplementary material for: Losing hope or keep searching for a golden solution: an in-depth exploration of experiences with extreme challenging behavior in nursing home residents with dementia
Source: BMC Geriatr. 2022 Sep 16;22:758. doi: 10.1186/s12877-022-03438-0 (PMC9479311; doi:10.1186/s12877-022-03438-0)
Supplement: Supplementary file 3 — Additional file 3: Supplementary material Table 3. Topic list for focus group discussions with interviewees. [file 12877_2022_3438_MOESM3_ESM.docx]

**Supplementary material Table 3. Topic list for focus group discussions with interviewees.**

| **Topic** | **Interview questions** |
| --- | --- |
| **Introduction**  **Treatment and care** | What is it that makes this situation a problem for the people involved?  What was the final point which made you all decide to involve the Centre for Consultation and Expertise (CCE)?  What difficulties did you experience during treatment of the resident’s extreme challenging behavior and in caring for the resident?  Which interventions were successful in treating the resident’s extreme challenging behavior and in caring for the resident?  Which interventions had no improvement in treating the resident’s extreme challenging behavior and in caring for the resident?  Why is/was the resident’s extreme challenging behavior untreatable/unsolvable? Do you have any idea about that? |
| **Multidisciplinary collaboration**  **Dealing with the situation** | How was the multidisciplinary collaboration in addressing this problematic situation?  What did you miss in the multidisciplinary collaboration in addressing this problematic situation?  What difficulties did you experience in dealing with the resident’s extreme challenging behavior?  What do/did you need in this kind of situation? |
| **Factors contributing to an impasse** | Do you think that a certain point of crisis was reached? (asked to all focus group discussion participants individually)  If yes, which factors contributed to this point of crisis?  At which point would you mark the situation as being a crisis?  What makes/made this situation different from other situations with residents with challenging behavior?  What has changed by involving the CCE? |
| **Impact of the resident’s extreme challenging behavior** | What was the impact of the resident’s extreme challenging behavior on you up until now?  Which emotions and feelings did the resident’s extreme challenging behavior evoke within you? |
