# Supplementary material for: Losing hope or keep searching for a golden solution: an in-depth exploration of experiences with extreme challenging behavior in nursing home residents with dementia
Source: BMC Geriatr. 2022 Sep 16;22:758. doi: 10.1186/s12877-022-03438-0 (PMC9479311; doi:10.1186/s12877-022-03438-0)
Supplement: Supplementary file 4 — Additional file 4: Supplementary Material Table 4. Additional quotes for all described general, main and sub-factors of the six (groups of) stakeholders. [file 12877_2022_3438_MOESM4_ESM.docx]

| **Supplementary Material Table 4: Additional quotes for all described general, main and sub-factors of the six (groups of) stakeholders.** | | | |
| --- | --- | --- | --- |
| **(GROUPS OF) STAKEHOLDERS** | **GENERAL AND MAIN FACTORS** | **SUB-FACTORS** | **QUOTE** |
|  |  |  |  |
| **RESIDENT** | **CHARACTERISTICS** |  |  |
|  | **PERSON** | Unlike other residents | Because he *(the resident)* has very good moments where... Of course, there will be a medical explanation for everything, but sometimes I really do feel and his wife says that too sometimes... Of course, the brain scan was not a full 100% because that was no longer possible because of his behavior. Yes, the other day she (*the relative)*  said: “He (*the resident)* still remembers everything sometimes. He is not really demented then.”  *(Case 3, Care staff member in focus group discussion)* |
|  | **BEHAVIOR** | Nature of the behavior | But aggression I do find, I do find that difficult, yes. You must also protect the fellow residents, and sometimes that is not possible at all. You are in a balancing act. You want to, but you just can’t.  *(Case 7, Care staff member in focus group discussion)* |
|  |  | Course of the behavior | Interviewer: “Why exactly was the situation perceived as a problem for you?”  I think the behavior is continuous. It's not intermittent but it is from the time when she *(the resident)* gets up to the time she goes to bed and wakes up again during the night, the behavior is continuous.  *(Case 4,Nurse in focus group discussion)* |
|  |  | Severity of the behavior | Sure, there are more residents with agitation, there certainly are, but not in that extreme form that this lady *(the resident)* has that it really goes on all night. Every night.  *(Case 5, Unit manager in focus group discussion)* |
|  |  | Unpredictability of the behavior | It *(the behavior)* comes all at once, it's unpredictable. I’ve had experiences with other residents that you can kind of sense that this aggression is coming on and with her *(the resident)*, you can't. It *(the behavior)* comes all at once and it may happen that she *(the resident)* walks over to you and at once she relaxes and is very sweet and quiet.  *(Case 7, Care staff member in focus group discussion)* |
|  |  | Unclear triggers of the behavior | What I see is a number of behaviors that I cannot properly pinpoint, I do not know what is behind them, and so I do not know what I need to treat.  *(Case 4,Elderly care physician in individual interview)* |
|  |  | Behavior considered as (partly) on purpose | She *(the resident)* did throw coffee all over someone once. Yeah, and then you’ll think, of course, she’s deliberately taking that cup and she’s deliberately throwing the coffee over someone... But whether it’s really conscious or just a scream for attention... Yes. Tricky.  *(**Case 7, Certified primary nurse assistant in individual interview)* |
|  |  | Behavior differs from personality before diagnosis of dementia | I also have the idea, at least from what I know of how she *(the resident)* is described by the family, that she is actually a very sweet gentle woman most of the time. So, the behavior she shows, which is also described here of suddenly being aggressive, to my mind it doesn’t fit the person she is.  *(Case 7, Elderly care physician in focus group discussion)* |
|  | **INTERACTION ISSUES WITH OTHER (GROUPS OF) STAKEHOLDERS** |  |  |
|  | **WITH OTHER RESIDENTS** | The resident’s behavior causes inconveniences and danger for the other residents | But also for the situation with the other residents to whom she *(the resident)* responds aggressively. And the other residents, they cannot act, they just have to take it. They cannot walk away, some are in a wheelchair but are still being beaten or almost pulled from the wheelchair, that too is, and not so much for the lady *(the resident)* or for the care staff, but perhaps is worst for the other residents.  *(Case 7, Care staff member in focus group discussion)* |
|  |  | Reactions of other residents negatively affect the resident’s behavior | And there’s a Turkish lady and she *(the resident)* absolutely doesn’t get along with her, you can’t leave those two alone. Because then things go wrong.  *(Case 7, Relative in individual interview)* |
|  | **WITH NURSING HOME STAFF** | The resident not understanding verbal requests | I definitely think that understanding is a factor. That she *(the resident)* really doesn’t know what... she can’t follow a conversation anymore... she also doesn’t know if you want to do something with her, help her, then she doesn’t know what you want either, you can’t explain that to her.  *(Case 7, Elderly care physician in individual interview)* |
|  |  | The resident giving short answers/minimal reaction | When you ask things, he *(the resident)* says very little to nothing. It is only “yes” or “no”.  *(Case 3, Certified primary nurse assistant in focus group discussion)* |
|  |  | The resident not wishing to/not making any contact | We do try to have a chat but he *(the resident)* very often indicates that he just doesn’t want to. Then he’ll keep his eyes closed too.  *(Case 3, Care staff member in individual interview)* |
|  |  | Inability of nursing home staff to read the resident’s emotions | It is sometimes a blank look in the eyes. That she *(the resident)* can look at you in a way of, like, what would I do? Sort of. But whether it *(the behavior)* is really fear then, or the lack of understanding I can’t, no... I find that a little difficult to say, what it is.  *(Case 7, Certified primary nurse assistant in individual interview)* |
|  |  | Nursing home staff not understanding the resident’s behavior and having no control over the behavior | And if she *(the resident)* is in an angry mood, then you can’t get her out of it either, you can try all sorts of things but then you can’t get her to calm down, she’ll stay in that mood.  *(Case 5, Certified primary nurse assistant in individual interview)* |
|  | **WITH CARE STAFF SPECIFICALLY** | Not noticing signs of escalation of the resident’s behavior in a timely manner | The last time he *(the resident)* grabbed me by my arm was when I wasn't focusing on him 100%, which meant I missed the signal. Yes, then I was too late in picking up the signal.  *(Case 3, Certified primary nurse assistant in individual interview)* |
|  |  | Positive moments with the resident are scarce | Yes, you know, then you really have to make the most of the good moments with the resident and if that is already very rare, then you don't get the positivity out of it anymore and then you remain very negative. Yes, and at one point, the whole care team, I understood that from the team meeting as well, the whole care team had actually just run out of steam.  *(Case 6, Certified primary nurse assistant in individual interview)* |
|  |  | Paying attention to the resident takes a lot of time | You actually have to divide five hours and 45 minutes among seven people *(other residents)*. But when I think about that, I think, my goodness, I spent two and a half hours on her *(the resident)*. And yes, if you add it up, I went along to the bathroom ten times that night. To her room, get some chocolate, walked into the living room together, sat together for a while, had a chat. Just to calm her down so that the other residents don't get restless as well, so that the other residents can watch television in peace for a while.  *(Case 4, Care staff member in individual interview)* |
|  |  | Undertaking pleasant activities with the resident is problematic | This man *(the resident)*, he was also cognitively deteriorating so it was increasingly difficult to go for a walk with him or to read together. Yes, that, that also decreased his quality of life.  *(Case 6, Unit manager in focus group discussion)* |
|  |  | Applying compulsory treatment is difficult | Yes, and that compulsory treatment, that’s something you’d rather not use. That is very unpleasant… It is very drastic for someone to do something to them against their will.  *(Case 1, Elderly care physician in training in individual interview)* |
| **RELATIVE** | **CHARACTERISTICS** |  |  |
|  | **PERCEPTIONS** | Having a different perception of the behavior, treatment and care | She *(the relative)* didn't want medication and she didn't think it was necessary, she had a very strong opinion about it… While it really wasn't possible at times.  *(Case 1, Unit manager in individual interview)* |
|  |  | Finding it hard to accept that usual care could not always be provided | Yes, his *(the resident’s)* wife had a really hard time with it in the beginning when he was incontinent and still wearing dirty clothes. Or that he had smears on his clothing and didn’t want a napkin. I think that we went along with that too much in the beginning.  *(Case 1, Care staff member in focus group discussion)* |
|  | **INTERACTION ISSUES WITH OTHER (GROUPS OF) STAKEHOLDERS** |  |  |
|  | **WITH NURSING HOME STAFF** | Nursing home staff insufficiently informs/involves relatives | It *(an intervention)* was not discussed with *(name relative)* or with another contact person, that is unfortunate... I think, to talk to the daughter sooner. I kind of missed that too. Kind of overlooked it myself.  *(Case 4, Certified primary nurse assistant in individual interview)* |
|  |  | Relative has limited trust in (certain) care staff members | I’ve had a lot of conversations with her *(the relative)* and she just doesn’t trust certain people. *(Case 1, Certified primary nurse assistant in individual interview)* |
|  |  | Relative criticizes actions of care staff | That we actually couldn’t do well in her *(the relative)* eyes no matter what we did... so I think it may have been a bit of self-preservation that caused her to react that way. But especially not doing it right in her eyes, that was difficult.  *(Case 1, Care staff member in individual interview)* |
|  |  | Relative crosses personal boundaries of care staff members | With a, a strong powerful wife who...  Yes, who also crossed our *(care staff)* limits.  *(Case 6, Unit manager and nurse in focus group discussion)* |
|  |  | Relative is ambivalent/uncommunicative about emotions and wishes for treatment | But I also find it very difficult to gauge them *(the relative and family)* in that respect, like, what they *(the relative and family)* really believe and what they really think.  *(Case 2, Certified primary nurse assistant in individual interview)* |
| **CARE STAFF** | **CHARACTERISTICS**  **PERSONALITY ISSUES** | Different approaches and interactions with the resident due to different personalities of care team members | She *(the resident)* has to deal with someone else every time, of course, who reacts differently to her. Of course, we are all different and everyone reacts differently  *(Case 4, Nurse in focus group discussion)* |
|  | **SKILLS ISSUES** | Having insufficient knowledge and experience | You don't learn these kinds of things *(About ways of dealing with the behavior)* in school. It gets mentioned once, people with problematic behavior, and you get a nice booklet. By the time it happens, you don’t remember.  *(Case 4, Certified primary nurse assistant in individual interview)* |
|  |  | Reports are of an insufficient quality | To put something about the behavior on paper... I find that a bit trickier myself and I think that is generally true... putting something clearly on paper is harder to convey than through a conversation.  *(Case 5, Care staff member on the nightshift in individual interview)* |
|  |  | Reflects insufficiently on own actions and feelings | That reflection *(of care staff members)* is so minor, it almost doesn’t happen. To express what it does to you exactly.  *(Case 4, Unit manager in individual interview)* |
|  | **ATTITUDE ISSUES** | Having a wait-and-see attitude/refraining from taking the initiative | Yes, because when you are indeed multidisciplinary, you still notice that the care staff is more of a follower. While I think, like, they are on it 24 hours a day. They are the ones that should lead.  And it is us *(the care staff)* that should actually say, like, listen, we have had it up to here, we need help now.  *(Case 1, Unit manager and nurse in focus group discussion)* |
|  |  | Not asking for help/asking for help too late | Yes, we didn’t show enough that we needed help.  *(Case 3, Care staff member in individual interview)* |
|  |  | Refraining from complying with the behavioral management approach that was agreed on | Yes, then if *(name psychologist)* gets involved and he prescribes advice, then we have to do that or try it. And then if that is eventually not done, it stops.  *(Case 4, Certified primary nurse assistant in individual interview)* |
|  |  | Having a fatalistic attitude | Then I had to do Mr… *(name resident)* again after a very long time. And then I was quite clearly told: “Yes, you have to be careful. Don’t stand on his right side. He hits and he does things”. So I went in there already thinking: Okay. ....That made sense, that they would warn you about that *(the behavior)*, of course. But I might have preferred to go back in with an open mind.  *(Case 3, Care staff member in focus group discussion)* |
|  |  | Differences in views on the behavior, approaches in dealing with the resident’s extreme challenging behavior and experiences of the behavior due to a difference in working shifts (day/night) and number of working hours | Being able to better understand the signals from colleagues sooner, because that's what it is… I am sometimes too easy when it comes to that. I then assume that others know and can do the same. And that is not so.  *(Case 3, Certified primary nurse assistant in individual interview)* |
|  |  | Difference in opinions about appropriate care | That’s what I found tricky too in terms of the approach, that you *(to a care staff member in the focus group discussion)* went and laid in her *(the resident’s)* bed with her *(the resident)* because that calmed her down. I thought that was really nice when I heard that and that you *(to a care staff member in the focus group discussion)* rocked her *(the resident)* so that she fell asleep, but *(name of another care staff member)* asks the question, and for good reason: isn't that too intimate? You are a professional.  *(Case 4, Psychologist in focus group discussion)* |
|  |  | Difference in the extent to which the resident’s behavior is accepted | Of course in one person the limit is here and in another the limit is there and I think that some persons kept providing care longer than they should have done, for their own sake.  *(Case 2, Care staff member in focus group discussion)* |
|  | **INTERACTION ISSUES WITHIN STAKEHOLDER GROUP**  **WITHIN CARE STAFF** | Little opportunity for formal and informal exchange of information | It’s difficult, because you don’t have many team meetings, you rarely sit down with your whole team together... an actual moment to talk about a case like this, we haven't really planned it.  *(Case 1, Care staff member in focus group discussion)* |
|  |  | Giving each other feedback is difficult | I find it difficult to confront a colleague with this. For I am afraid I’ll have a discussion or get a nasty reaction. And I don’t want that. I want to avoid that.  *(Case 3, Care staff member in focus group discussion)* |
|  |  | New ideas from care staff members often receive a negative response from other care staff members | In any case, it is true that there is also someone in that team *(care staff)* with a special focus on people with problematic behavior and that someone has taken a special course for that. And they tried to explain a little bit about it, but it didn’t go down well with the rest of the team, at least not with everyone in that team. So there’s some, yes, I did sense some friction there... you think you know it all, but we actually know it too and we do it this way.  *(Case 4, Elderly care physician in individual interview)* |
|  |  | Communication takes place indirectly | Look, one week I see  *(name of a care staff member)* very often, and then, just like that, I don’t see her for two, three weeks. Look, it also just depends on when someone works... And when I discuss things with her, they don’t all know...  *(Case 1, Care staff member in focus group discussion)* |
| **TREATMENT STAFF** | **CHARACTERISTICS** |  |  |
|  | **BEING AT BAY** | Missing the whole picture of the  situation and the resident’s behavior | He *(the elderly care physician)* only sees part of her *(the resident)* sometimes. I find that situation very difficult.  *(Case 2, Certified primary nurse assistant in individual*  *interview)* |
|  |  | Only present during office hours | If you are in large-scale unit, then you are in your office in the middle of the department. Then you don't miss anything. In small-scale units, you really are not involved. And that takes some getting used to. They *(the care staff)* really have to make an effort to tell me something.  *(Case 1, Unit manager in individual interview)* |
|  | **TREATMENT ISSUES** | Difficult to develop and implement a  treatment plan | It’s everything, the whole picture. It's not one thing and that's how you solve it.  *(Case 3, Certified primary nurse assistant in individual interview)* |
|  |  | Treatment plans have no  effect/temporary effect | Well, we have done so much and tried so much and it has all either helped temporarily or not at all.  *(Case 2, Care staff member in focus group discussion)* |
|  |  | The situation often needed to end as  soon as possible | And then the medication is changed and then you hope that the behavior changes too... You have to give that another couple of weeks and then it still does not work adequately, leading to it being changed again. It then often takes another few weeks to give the medication a chance... of course you just don’t want that behavior, you want it gone as soon as possible. But by giving the medication a chance you have to, yes, that just takes a long time.  *(Case 6, Certified primary nurse assistant in individual interview)* |
|  |  | Difficulties with prescribing medication | It was particularly intense at first when she *(the resident)* had just been admitted, when Ms … (*name of the resident)* would walk around the unit, banging on the windows that she wanted to go home. That, before, you were just putting out fires and then we would intervene with medication to de-escalate... it was actually intervening immediately to get things under control.  *(Case 4, Elderly care physician in focus group discussion)*  The PIT team *(advisory team)* came up with the advice: yes, cut down that medication, because it doesn’t do anything. So we cut it down and everything was derailed.  *(Case 2, Elderly care physician in individual interview)*  I would personally feel very bad, that the man *(the resident)* is sitting in the chair completely dazed, that it doesn't do his wife any good. That it doesn't do him any good. And that should not be at the expense of all of us. So, that should actually balance out a little bit. But, well, there isn’t a balance, really.  *(Case 3, Care staff member in focus group discussion)* |
|  | **SKILLS ISSUES** | Having insufficient knowledge and  Experience | Lack of experience with extreme behavior. This also makes it difficult for me to treat... I don’t have the knowledge and experience to deal with it *(the behavior)*.  *(Case 2, Psychologist in individual interview)* |
|  |  | Making treatment plans which are  outdated/ impractical/unachievable/not  feasible | *Name* *of behavioral expert* is a behavioral expert, who has identified a lot of good things and made very nice diagrams of what you can do and what you cannot do and how should you do it. But well, that, you know in advance that it doesn’t help.  *(Case 4, Certified primary nurse assistant in individual interview)* |
|  |  | Unable to detect the needs of the care  staff, meet their expectations or support  them properly | I think we needed a little more guidance, in terms of the care staff. From her *(the psychologist)* there could have been a little more support, more meetings with us.  *(Case 1, Certified primary nurse assistant in individual interview)* |
|  |  | Involving external expertise too late | Well, some actions could have been faster, I can tell you that. Yes, that intervention team could have been involved a little sooner. That may have been delayed for too long, even by myself. And the Centre for Consultation and Expertise (CCE) could have been involved more quickly, too. Also the psychiatrist in consultation. It could all have been a bit faster.  *(Case 2, Psychologist in individual interview)* |
|  | **ATTITUDE ISSUES** | Being indecisive/taking little  Responsibility | She *(the psychologist)* didn’t feel it was her responsibility to do anything about it. Yes, it was within her capabilities, but that was insufficient in this situation, there was just more to be done and that, she didn’t feel that was her responsibility.  *(Case 3, Unit manager in individual interview)* |
|  |  | Undertaking too few actions | If you look at it in terms of actual actions, very little has happened.  *(Case 6, Unit manager about elderly care physician in individual interview)* |
|  |  | Not informing themselves properly about the (severity of) the behavior | Being a little more on top of things, that they *(the treatment staff)* come round more often...  *(Case 1, Care staff member in focus group discussion)* |
|  |  | Unaware of the expertise of care staff | That I sometimes notice, that’s not to be mean, but they *(the treatment staff)* then come to us to start such a plan, but then it actually has to come from us *(the care staff)*, sure we are the experiential experts.  *(Case 7, Certified primary nurse assistant in individual interview)* |
|  | **INTERACTION ISSUES WITHIN STAKEHOLDER GROUP** |  |  |
|  | **WITHIN TREATMENT STAFF** | Different perceptions as to everyone’s responsibilities pertaining to the situation | They *(about the elderly care physicians in general)* say, yes, well, this is a solution and you are in charge of the organisation, so go and arrange it… I think that's a bit too easy… and maybe I'll favour it too, but then there has been a doctors meeting and then everything is discussed and then you get an email. *Unit manager's name*, do you want to take care of camera surveillance, do you want to take care of this... Sometimes they say: just arrange some staff for it. Well, and then it is put on my plate, like, well, you sort it out. Please, and then I think, yeah, right, how nice.  *(Case 1, Unit manager in individual interview)* |
|  |  | Not enough formal and informal exchange of information between the psychologist and elderly care physician | She *(unit manager)* works a lot with the elderly care physician, so with that transfer they *(elderly care physician and unit manager)* have pretty much ignored me for example, so to speak.  *(Case 7, Psychologist in individual interview)* |
|  |  |  |  |
|  |  |  |  |
| **NURSING HOME STAFF** | **INTERACTION ISSUES**  **WITHIN STAKEHOLDER GROUP** |  |  |
|  | **QUALITY OF INTERDISCIPLINARY COMMUNICATION** | Limited exchange of information due to few meetings | And perhaps you should also sit down together as a team sooner, as a kind of multidisciplinary team meeting, and ask, how do we deal with this.  *(Case 4, Care staff member in focus group discussion)* |
|  |  | No room for reflection | I do think that we need to have a little more consultation with each other, like when do you get stuck and how do we get stuck? What are your issues, I don't think we have always expressed that properly to each other.  *(Case 4, Elderly care physician in focus group discussion)* |
|  |  | No room for giving each other feedback | Giving feedback or anything together, that’s difficult anyway, let alone to someone who... is in another discipline who is a little better educated.  *(Case 1, Care staff member in focus group discussion)* |
|  |  | No room for an extensive analysis of the behavior | Then, I think, you do have to look at everything much more broadly, much earlier... I think that not everyone was aware of the resident's history, his life and pathology, what this entails and what kind of behavior  comes along with it.  *(Case 3, Certified primary nurse assistant in individual interview)* |
|  |  | Care staff members not communicating their needs, wishes and actions taken with the treatment staff | They *(about the care staff)* are really good at a lot of things, but even then saying, like, we are working on that, is a problem, though.  *(Case 1, Unit manager in focus group discussion)* |
|  |  | Care staff members share incomplete and unclear information | Interviewer: “Are you saying that the care team did not clearly present the problem to the elderly care physician? Or that the elderly care physician didn’t see the seriousness of the situation. Why is that, do you think?” For a small part, that has to do with the clarity of communication of the team.  *(Case 7, Unit manager in individual interview)* |
|  |  | Treatment staff members insufficiently involving care staff in their plans | Maybe we should have coordinated a little more with, with the care team.  *(Case 4, Psychologist in focus group discussion)* |
|  |  | Care and treatment staff not taking each other seriously or not listening to each other’s ideas/rationalizations for approaching the problem | Then I noticed that there were annoyances there and the care staff said, we don’t feel heard, while *(elderly care physician's name)* said, you don’t agree with my decision and that’s what makes you react like that. So that was the end of the moral deliberation, especially that they would listen to each other more and why do you do it this way with Ms (*resident’s name)* and what is the underlying thought, there was no listening to that at all by the team.  *(Case 2, Unit manager in individual interview)* |
|  | **INEFFICIENT WORK PROCESSSES** | Indirect communication between care and treatment staff | If you have an issue now and you run into something, say... a notebook, and I respond to it and I look at it and I turn the notebook back in and done. I think you need to start making much more use of each other’s disciplines. Like, what is your disciplinary input and what exactly do you run into? It’s not a matter of questions and answers and done. That is not how it works.  *(Case 4, Elderly care physician in focus group discussion)*  They *(about care team on night shifts)* are also part of another team so they have their work meetings there, that goes via other routes... that soon makes the consultation indirect. That goes, for example, for *the unit manager's name*, it goes for a number of people, but also for some of her colleagues, so it’s often indirect.  *(Case 5, Elderly care physician in individual interview)* |
|  |  | Inefficient communication due to a missing working agreement | Then, for example, I have to visit a resident where I discuss the misunderstood behavior with a care staff member and right after my visit there’s an appointment with the psychologist where that same misunderstood behavior is discussed and I think that twice over is a bit much.  *(Case 5, Elderly care physician in individual interview)*  We actually have very little contact, especially as care staff members, we have very little contact with the psychologist. That all actually runs via the certified primary nurse assistant ... so that you are hardly in the know as a team.  *(Case 1, Care staff member in focus group discussion)*  But what happens *name of care staff member* can stand this behavior well, *name of another care staff member* cannot stand this behavior, but when *name of care staff member* has a visit of the elderly care physician, then according to *name of another care staff member* the behavior comes across differently.  *(Case 7, Unit manager in individual interview)*  And then if something is needed to be discussed, the psychologist would  discuss it with the intern. Well, I think that is wrong and then I confront them about it. I am the care provider, at that time I am ultimately responsible for the care of my residents and that you don’t consult with an intern.  *(Case 3, Care staff member in individual interview)* |
| **ORGANIZATION** | **CHARACTERISTICS** |  |  |
|  | **STAFFING ISSUES** | Short staffing and staff-turnover | Very soon after I started in February, March, I very quickly got the question why do we have so few staff, we really have a shortage of staff, we have a resident in our group who demands a lot of attention and as a result we can no longer provide the other residents with quality care.  *(Case 4, Unit manager in individual interview)*  There are some staff changes. Of course, it’s a unit with a great numer of staff anyway... so he *(the resident)* does see a lot of different people.  *(Case 3, Unit manager in individual interview)* |
|  |  | Excessive workload | Yes, then you have to run sometimes, because you have seven people in the group. You actually have to divide five hours and 45 minutes among seven people. But when I think about that, I think, my goodness, I spent two and a half hours on her.  *(Case 4, Care staff member in individual interview)* |
|  | **UNIT** | Size of the unit | The environment doesn't work in my favour, because it continues to be busy in the unit, I can’t make it quieter there. There are really far too many people living here in too small an area.  *(Case 6, Unit manager in individual interview)* |
|  | **ORGANIZATIONAL NORMS** | Acceptance of the behavior by considering it as part of the dementia or the resident’s personality | If you yourself don't think the behavior is actually normal, you don't dare say it. Another says you work with this target group for a reason, that *(behavior)* is normal, and I then say that is not normal... I think it is the culture... the culture in the team makes it so that no-one dares to be so open, like, where is my limit?  *(Case 4, Unit manager in individual interview)* |
|  | **ROLE OF MANAGEMENT** | Management staff insufficiently investing in solutions to improve the situation for the resident | What I have missed in the whole story is that *the name of the institution* would put some money into Ms’s  *(the resident’s name)*... room. Other curtains to add depth, stick a front door on her door, an alarm with snooze, a little more... Yes, just name everything you can think of and I miss it. *(Case 4, Certified primary nurse assistant in individual interview)* |
|  |  | Management staff making decisions interfering with the clinical situation | It has also saddened me as a person and especially because the management team interfered with the content... I really did have very unpleasant weekends because of that... what they do closes the door to my relationship with the relatives.  *(Case 6, Unit manager in individual interview)* |
